# Particle–Nanofiber Superstructures

**DOI:** 10.1021/accountsmr.5c00308

**Published:** 2026-04-21

**Authors:** Bin Zhao, Luiz G. Greca, Bruno D. Mattos

**Affiliations:** † Department of Applied Physics, School of Science, 174277 Aalto University; Espoo FI-00076, Finland; ‡ Department of Bioproducts and Biosystems, School of Chemical Engineering, 174277 Aalto University; Espoo FI-02150, Finland

## Abstract

Superstructured particle assemblies are sought as efficient platforms for transferring properties from the nano- to macroscale while combining modularity and versatility with facile and scalable fabrication processes. Such assemblies are achieved by structuring primary particle using various assembly techniques, which enables the control and customization of morphological features across length scales. Ensuring high cohesion within these assemblies is crucial for practical applications, both to mitigate nanotoxicity and bioaccumulation and to prevent the loss of performance resulting from subunit detachment. In this Account, the integration of biobased nanofibers into particle constructs is discussed in terms of their ability to act as universal binders that offer several advantages over case-specific strategies to develop strength in superstructures. Cellulose nanofibers, among others, have a remarkable capacity to confer cohesion to virtually any particulate system, thereby improving strength and toughness and opening several opportunities to manipulate their nano- to macrostructures. At the nano- and microscale, nanofibers can disrupt particle lattices, thereby enhancing access to surface functionalities. At the macroscale, nanofibers enable control over the viscoelastic properties of particle suspensions and govern their consolidation into dried particle–nanofiber constructs. Cellulose nanofibers are the most widely used in supraparticle fabrication, but several other fibrillar nanomaterials from chitin, amyloid, and aramid show promise for a broad range of particle–nanofiber assemblies. This Account presents a detailed review of the ability of nanofibers to enhance cohesion and manipulate supraparticle structures. First, cellulose nanofibers are introduced, and aspects like extraction, surface chemistry, modification, and colloidal properties are discussed, as they play important roles in transferring cohesion from the nanofibrillar network onto the particle–nanofiber assembly. Then, nanofiber–particle interactions in dilute and concentrated regimes are discussed and related to the forces and phenomena that drive the consolidation of these robust constructs. Analyses of mesh size and crowding of associated nanofiber networks are put into perspective and discussed in terms of particle entrapment and particle–nanofiber interactions prior to consolidation. Methods currently employed to fabricate superstructured materials are presented, including casting on superhydrophobic surfaces, templates and hydrophilic substrates, foaming for particle–nanofiber networks at air–liquid interfaces, 3D printing, and spray drying. Finally, practical applications for particle–nanofiber superstructures are introduced and discussed in terms of the gains obtained by using well-defined nanostructured materials and supraparticles.

## Graphical abstract

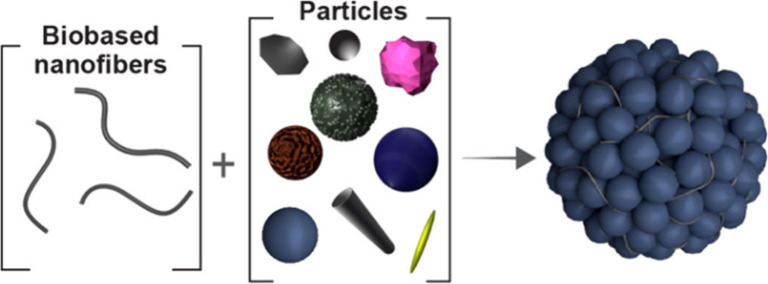

## 1 Introduction

Superstructuring particles across length scales is an effective way to fully exploit the unique functionalities of nanoparticles in macroscopic materials or systems.^1,2^ Superstructured particle assemblies combine modularity and versatility in terms of formulation with facile fabrication methods.^3,4^ Following simple drying processes, precursor particle suspensions are consolidated into supraparticles (SPs) ranging from a few micrometers^5^ to several millimeters in size.^6^ SPs have demonstrated multiple applications, where the interplay of the primary building blocks, packing-porosity, and macroscopic size and morphology yields a broad range of tailorable properties. Maintaining robust cohesion within particle assemblies is essential for their reliable implementation in applications, as insufficient integrity can lead to nanotoxicity, bioaccumulation, and performance loss stemming from the release of elementary subunits.^7^ In particles of relatively small radii, interparticle cohesive contact forces far surpass the gravitational forces of the individual subunits, thus yielding superstructures with mechanical integrity.^7^ However, interparticle contacts scale inversely with the cube of the particle diameter, leading to a rapid reduction in construct cohesion when the primary particles are in the submicrometer to micrometer size range. Besides pure dimensional features, interparticle forces vary according to the particle surface chemistry^8^ and processing conditions employed during their assembly.^9,10^ Overall, cohesion in particle assemblies is approached in a case-by-case fashion, mostly depending on the particle of interest.

The use of biobased nanofibers as minor additives substantially enhances the cohesion of superstructured constructs while providing a versatile toolbox for finely tuning their nano- and macrostructures. At the nano- to microscale, nanofibers can induce disruption in the particle network, thereby increasing access to surface-derived functionalities. At the macroscopic length scale, nanofibers can be used for tailoring the SP morphology by influencing the kinetics of consolidation from a suspension droplet into a dried construct. Cellulose nanofibers, obtained from the mechanical defibrillation of hierarchically structured plant fibers, were the first to be used in SPs due to their high strength (3 GPa ultimate strength and 40 GPa elastic modulus), high aspect ratio (up to 100), branched morphology, and high flexibility (smallest rigid units of 150 nm) that allows it to bend and network around particles of small radii of curvature. The successful integration of cellulosic nanofibers in particle constructs has inspired the more recent use of proteinaceous amyloid fibrils in supraparticle fabrication.^11^ This demonstrates the transferability of the proposed nanofiber-centered framework for manipulating SP properties to other nanofibers, processing conditions, as well as applications.

This Account reviews the capacity of nanofibers to induce strong cohesion in particle assemblies while providing a versatile toolbox for tailoring the structure from the nano- to macroscale. It first introduces key aspects of the extraction and chemical modification of cellulose nanofibers and discusses how these factors govern their networking capabilities and surface chemistry, both of which critically influence particle binding. The discussion proceeds from nanofiber networks in dilute and gelled systems to the development of capillary forces during consolidation, eventually leading to the formation of dried nanofiber–particle constructs. Particular attention is given to the main physical frameworks used to describe nanofiber–particle processing, namely, network mesh size and crowding factor. Practical aspects of nanofiber–particle systems, including fabrication methods and associated applications, are subsequently reviewed. The Account concludes with an outlook on the challenges and opportunities associated with extending this approach to other classes of technologically relevant nanofibers.

## 2 Biobased Nanofibers and Their Relevance to Superstructures

In the context of superstructures, nanofibers are sought as structural binders that can introduce high cohesion in particle constructs at minimal loadings (1–15 wt %) while maintaining access to the particle functionalities. Cellulose nanofibers are the most employed structural binders in particle superstructuring, while amyloid nanofibers have been used in cases where bioaccumulation could occur.^11^ Positively charged chitin nanofibers are also used for assembling superstructures, as they provide surface chemistries not naturally present in plant-based counterparts.^12^

Biobased cellulose nanofibers have high aspect ratio (up to 100), flexible (minimal rigid segment of ∼100–150 nm), strong (*E*_*A*_ up to 100 GPa), and often branched building blocks, which are ideal features to superstructure particles solely via nanonetworking. Cellulose nanofibers form strong gel networks at low mass fractions (≤1.5 wt %), enabling the physical entrapment of particles into robust, homogeneous (no phase separation) constructs. The degree of nanofiber entanglement is critical for transferring cohesion from the network to the resulting supraparticles, while the surface chemistry and morphology of nanofibers govern their assembly behavior and interactions with particulate matter. Both properties are determined by the chemical composition of the fiber precursors and by modifications introduced during nanofiber isolation. Plant-based nanofibers can be extracted from virtually any higher plant (Figure 1a), but the cellulose content as well as the biomass abundance (Figure 1b) must be considered for feasibility purposes. Wood is highly abundant, and its relatively high content of cellulose puts such biomass in the forefront of academic and industrial efforts related to cellulose nanofibers.^13^

**Figure 1:**
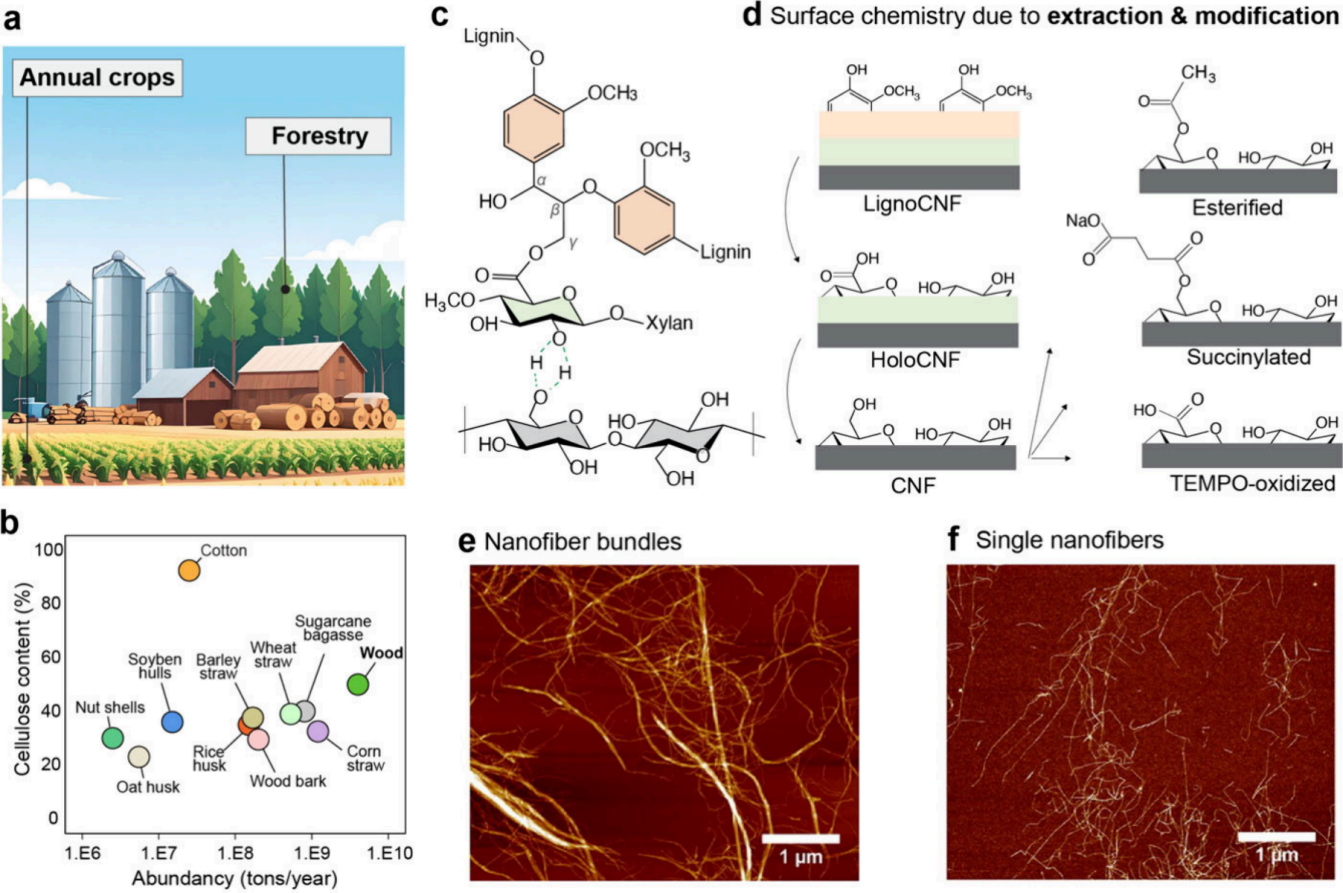
(a) Nanofibers can be extracted from both annual crops and forests. (b) Biomass can be converted into cellulose nanofibers, which are exceptionally abundant in nature (values in (b) are approximations based on databases from FAO, Embrapa, International Nut and Dried Fruit Association, and other institutes in the area). (c) Plant biomass is primarily composed of cellulose, hemicellulose, and lignin. (d) The surface chemistry of the resulting nanofibers depends on the degree of biorefining (left column) as well as modifications during nanofiber preparation (right column). (a–d) Created by B.D.M. (2026). Plant nanofibers can be extracted as (e) nanofiber bundles or (f) single nanofibers, depending on the defibrillation methods taken. (e, f) Reproduced with permission from ref 19. Copyright 2014 American Chemical Society.

In higher plants, cellulose fibers are bound together by lignin via hemicelluloses. Whereas hemicelluloses interact with cellulose via supramolecular interactions, they are often covalently bound to lignin via lignin–carbohydrate complexes (Figure 1c). Depending on the deconstruction degree of the starting precursor, the resulting nanofibers can contain drastically different surface chemistries (Figure 1d, left) and morphologies. Although native cellulose nanofibers (CNFs) are the most employed structural binders (Figure 1e), there are several efforts toward exploiting their tailored surface chemistry. For example, hemicellulose-containing (HoloCNF)^14^ and lignin-containing nanofibers (LignoCNF)^15^ could play a better binding role in particle constructs of highly hydrophilic components or in those aiming at carbon materials, respectively. Tannin-containing cellulose nanofibers are effectively obtained by cogrinding defibrillation.^16,17^ Nevertheless, such tannin-containing nanofibers could, for example, enable the formation of hybrid materials where metal–phenolic networks^18^ are homogeneously distributed in the superstructures.

Nevertheless, a plethora of modification pathways to modify cellulose at bulk and surface levels have been demonstrated. Some modifications, including TEMPO-oxidation^20^ and regioselective and reversible succinylation,^21^ induce high charges (carboxylate content of 1.5 mmol/g) at the fiber level so that the subsequent mechanical defibrillation leads to the isolation of single nanofibers (Figure 1f). Therefore, both the morphology and the surface chemistry can be tuned in cellulose nanofibers for optimal particle binding.

## 3 Overcoming Particle Dependency with Nanofibers

When supraparticles (SPs) are fabricated without nanofibers, cohesion is introduced in a case-by-case fashion, using, for instance, surface modification^17^ or sintering.^22^ Nanofibers can bind virtually any type of particle, restrict their mobility, and consolidate them into extremely robust assemblies, regardless of their surface chemistry, composition, geometry, or size. This Account focuses primarily on cellulose nanofibers (CNFs), representing the most employed structural binders. In general, single nanofibers are highly charged (0.5–1.5 mmol/g) and gel at much lower concentrations (∼3 g/L) than typically noncharged branched or bundled nanofibers, which gel only at ∼10 g/L.^13,23^ Moreover, single (smaller) nanofibers are likely better candidates for (i) binding smaller particles due to the optimal size relationship and (ii) superstructuring particles in size-constricted systems such as microfluidics. Given the broad variety of cellulose nanofibers, understanding how entangled networks and particles interact in suspension is therefore the key to engineering colloidal binders.

### 3.1 Nanofiber–Particle Interactions in Suspension (Dilute Regimes)

Particle–nanofiber materials typically start with dilute precursor suspensions to obtain a homogeneous dispersion of the two (or more) components. Upon mixing (e.g., by sonication or vortex), hard spheres can push fibers around and accommodate themselves among the fibrillar network. The state of the suspensions soon after mixing can critically affect the topology of the final constructs.^24^

For nanofibers and particles displaying low charge density, particles move freely within the fiber network if their size is smaller than the mesh size. Generally, this makes them more susceptible to diffusion and drying effects. Mesh formation (its characteristic size) plays a key role in enabling nanofiber networks to cage particles under dilute conditions. A dimensional relationship between mesh size and particle diffusion has been established (Figure 2a–c), and it can be used later to engineer several superstructured materials. Nevertheless, we recognize the existence of several other approaches and models to analyze particle diffusion and mobility in polymer networks, with modes that are intermediate to free moving and caged, such as anomalous and hopping diffusion.^25^ Besides Stokes–Einstein, other models used for polymer networks (e.g., Schweizer) should eventually be utilized to further understand nanofiber–particle systems.^26^

**Figure 2:**
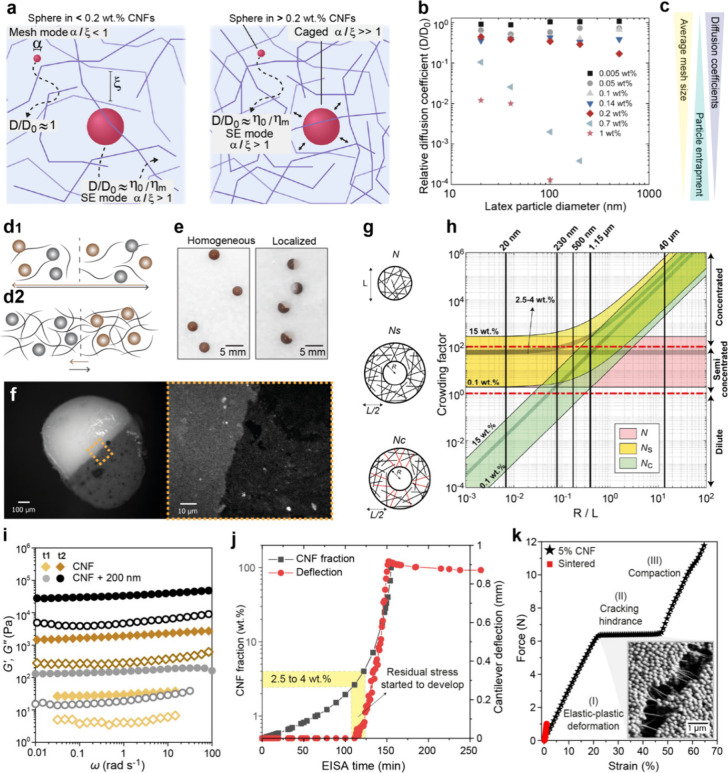
Fundamental aspects related to particle–nanofiber interactions in suspension. (a) Analysis of mesh size versus particle mobility, displaying key conditions for the particles to move freely (Stokes–Einstein, SE) or to be caged among fibrils. (b) Relative diffusion coefficients of particles as a function of the particle diameter in CNF dispersions. (a, b) Reproduced with permission from ref 27. Copyright 2022 The Authors. (c) Simplified relationship among mesh size, particle entrapment, and diffusion coefficients. Created by B.D.M. (2026). Schematics of conditions where particles can freely move (d_1_) or are immobile (d_2_) at interfaces of two droplets containing nanofibers and particles (also visually seen in e). () Resulting supraparticle with hemispherical domains obtained from silica and iron oxide suspensions, at 5 wt % CNF content. Note: Images from (d) to (f) are unpublished results from the authors. (g) Schematic representation of the traditional crowding factor (*N*) and its derivation into the surface crowding factor (*N*_*s*_) and contact crowding factor (*N*_*c*_). (h) Theoretical plot that numerically describes *N*, *N*_*s*_, and *N*_*c*_ for nanofibers and particles of varied sizes. (i) Viscoelastic properties of suspensions containing cellulose nanofibers and silica particles measured at the beginning (t1) and after evaporation-induced self-assembly (t2). Note that concentrations are 0.75 wt % CNFs and 5 wt % particles at t1 and 3 wt % CNFs and 20 wt % particles at t2. (j) Plot of the development of residual stresses, by the cantilever deflection method, correlated with the increase in CNF mass fraction due to EISA. (k) Typical mechanical behavior of nanofiber-bound supraparticles. (g–k) Reproduced with permission from ref 4. Copyright 2020 The Authors.

By having a slow drying rate, e.g., through evaporation-induced self-assembly (EISA) under ambient conditions, an optimal balance can be achieved to form supraparticles of largely uniform composition and topology. Limiting particle diffusion is also useful for the design of multicomponent systems. For instance, supraparticles can be engineered with two hemispheres of distinct compositions (Figure 2d–f). In one demonstration, two suspensions with the same CNF content, with one containing silica and the other containing iron oxide particles, were cast side by side onto a superhydrophobic surface. Upon contact, two droplet suspensions merge instantly into a homogeneous, larger droplet when the CNF concentration is 2% or less (Figure 2d_1_). In contrast, at 5% CNFs, a distinct interface forms and is retained throughout the consolidation (Figure 2d_2_).

In systems comprising oppositely charged species, particles bind to nanofibers and remain kinetically trapped at fixed positions relative to the nanofiber network. This happens even when the particle size is smaller than the mesh size. If the suspension is not dilute enough, strong complexation can occur.^24^ This is detrimental to achieving a homogeneous distribution of particles and may be difficult to overcome via mixing. Therefore, properly accounting for such interactions and their macroscale effects is an important aspect in the design of diverse superstructured constructs.

Lastly, fiber interactions have been widely analyzed using the crowding factor (*N*), which is a coefficient traditionally used for analyzing the percolation of fiber networks. The crowding factor has been recently correlated with particle diffusion across various nanofiber mesh sizes. Although the correlations follow rational trends, i.e., particle mobility correlates inversely with *N*,^27^ the traditional coefficient underestimates nanofiber population around the particles given the volumetric constraints. It also fails to quantify nanofiber abundance in the interparticle region or the probability of nanofiber–particle surface contacts, both of which are critical for forming cohesive particle assemblies. Accordingly, surface (*N*_*s*_) and contact crowding (*N*_*c*_) factors have been introduced to describe the local population of nanofibers surrounding particles and the probability of nanofiber–particle contact interactions (Figure 2g). With *N*_*s*_ and *N*_*c*_, crowding is not only a function of nanofiber dimensions; it also considers the effective dimensional relationship between particles and nanofibers, thus providing a more insightful set of numerical information, especially to analyze fundamental aspects related to particle–nanofiber interactions from dilute to concentrated regimes (Figure 2h). For a deeper understanding and access to the new equations of such an analytical framework, we recommend readers to see our earlier work.^4^

### 3.2 Particle–Nanofiber Interactions during Consolidation

Particles and nanofibers strongly interact only during the late stages of consolidation. The viscoelastic properties of particle–CNF suspensions do not drastically change before approaching the gelation point (Figure 2i), although suspensions of particles with aspect ratios diverging from 1 (e.g., platelets and rods) tend to form complex microstructures that alter their viscoelastic properties near gelation.^28^ Very importantly, nanofiber suspension gels at much lower solid fractions than spherical particle suspensions, which is key to entrapping particles across the particle–nanofiber superstructure. During the final stage of particle–nanofiber supraparticle formation, consolidation is predominantly driven by drying stresses that emerge when water evaporates from the gelled suspension (Figure 2j). Residual stresses arising during drying have been evaluated by using the cantilever method, and their relationship to nanofiber–particle gelation and consolidation has been elucidated. Clearly, for cellulose nanofibers and hydrophilic silica, residual stresses commence almost instantaneously at the gelation point of the suspensions (Figure 2j). As free water is removed from the system, high capillary forces bring particles and nanofibrils into proximity and maximize short-range interactions (Figure 3a). For hydrophilic components, these are mainly H-bonds and van der Waals interactions, but they can largely differ based on surface chemistry (Figure 3b).

**Figure 3:**
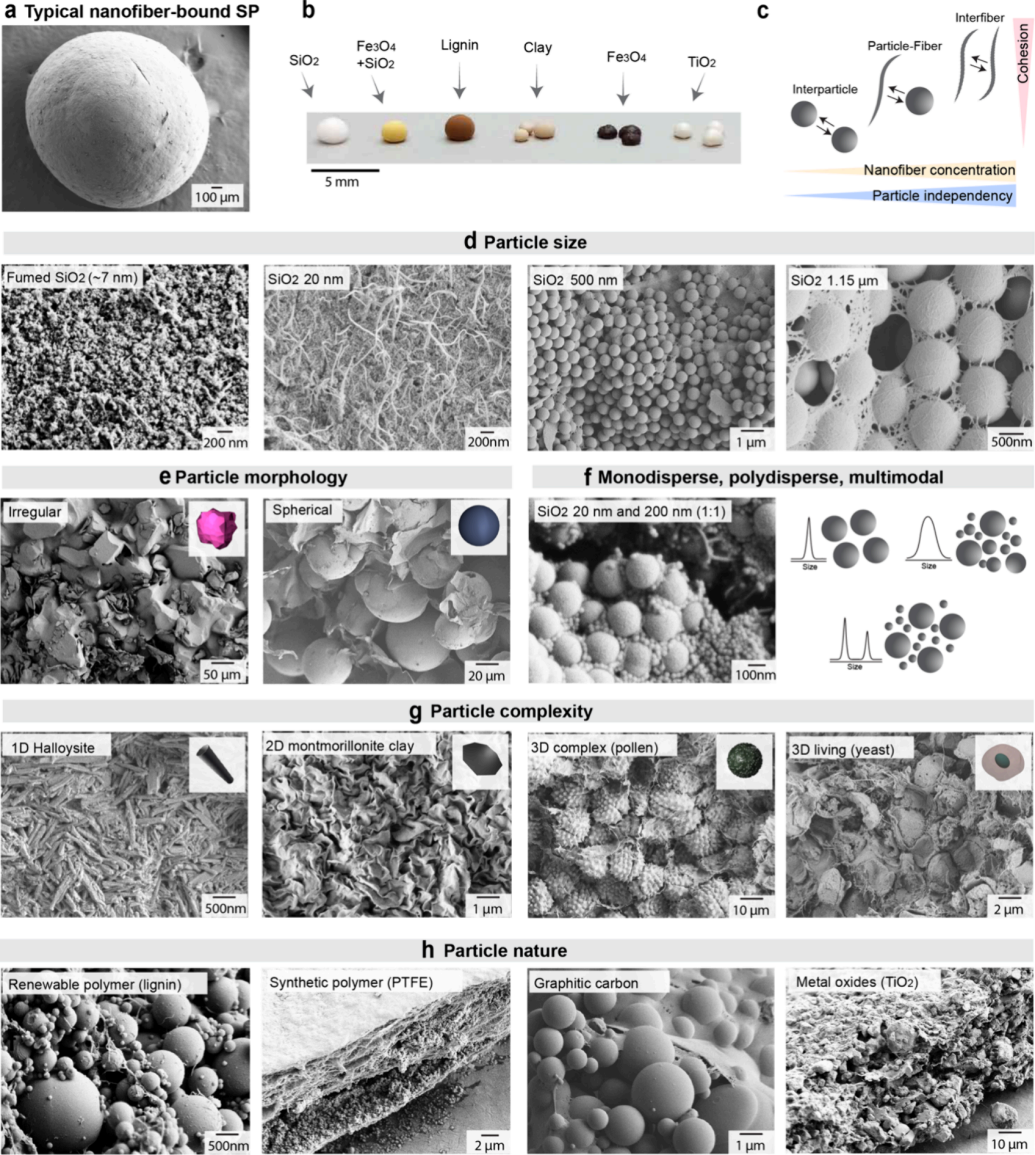
Library of superstructures obtained from cellulose nanofibers and various particles. (a) Typical nanofiber-bound SP and (b) examples of SPs with varied primary building blocks. (b) Reproduced with permission from ref 4. Copyright 2020 The Authors. (c) Sufficient nanofiber–nanofiber interactions lead to universality in particle binding by CNFs. Created by B.D.M. (2026). CNFs have been used to bind (d) particles of various sizes; (e) spherical or irregularly shaped particles; (f) monodisperse, multimodal, and polydisperse particles; (g) particles of various complexities including tubes, platelets 3D hedgehog-like, and living; and (h) particles of various natures, including metallic, ceramic, and polymeric. (a) and (d–h) All microscopy images belong to the personal archive of the authors.

For monodisperse small hydrophilic particles (e.g., 20 nm SiO_2_), the particle–nanofiber network is homogeneous, with the particles uniformly distributed and embedded within the nanofiber network (Figure 3c). At this dimension, the nanofibers are too rigid to make conformal contact around the small nanoparticles. Such small particles also end up acting as spacers, preventing the development of optimal nanofiber–nanofiber contacts. Although the interaction of a nanofiber with multiple particles brings cohesion to the construct, this topology is still not optimal. For medium-sized particles (from ca. 230 nm to 1.15 μm), nanofibers effectively conform and wrap around the particles in the form of a thin network. This final nanofiber arrangement, largely reminiscent of capillary bridges formed at late stages of consolidation, results in optimal particle–nanofiber and nanofiber–nanofiber contact and consequent mechanical properties. For large particles (ca. 40 μm), the nanofibers primarily accumulate at the void spaces and form a cellular architecture enveloping the large particles. Here, a major portion of the nanofibers consolidate into sheet-like structures, where nanofibers interact mainly with each other. This arrangement results in a similar cohesion to that observed for small nanoparticles.

Beyond particle size, surface chemistry profoundly influences supraparticle topology and its mechanical performance (Figure 3b). When model hydrophobized silica particles are used, hydrophobic interactions in suspension lead to the formation of large (ca. 10 μm diameter) particle aggregates that are largely unaffected by drying forces. Particularly, these aggregates are enveloped by a cellular type of fibrillar network akin to the topology observed in systems having large silica particles. With positive surface charges (e.g., amino-functionalized silica particles), strong fibril–particle interactions promote the formation of ionic complexes that lead to smaller (ca. 1 to 3 μm) particle–nanofiber aggregates bound within a nanofiber network.

In general, particle–nanofiber supraparticles are much stronger and tougher than their pure particle counterparts (Figure 2k). To exemplify this, we highlight an SP made of 200 nm SiO_2_ particles without and with only 5% of cellulose nanofibers. Adding nanofibers introduces higher cohesion in the overall superstructures, as indicated by a pronounced extension of the elastic-plastic regime (I) of the force–strain curve. After reaching the yield point, rather than breaking, as is typical for particle-only SPs, the nanofiber-containing SPs display a typical plateau zone (II) that indicates reinforcement against cracking. This is nicely visualized in the inset SEM image in Figure 2k. A densification regime (III) still takes place, and the SPs never fully break and cause unitary particles to detach. These three structural-centered mechanisms are seen across all nanofiber-containing SPs, regardless of the final topology of the nanofiber network around particles. The SPs showed strong resistance to simulated environmental stresses, including moisture, temperature, mechanical loading, and corrosion.^6^ Fully wet SPs were significantly weaker, likely due to reduced fiber entanglement and fiber–particle interactions upon cellulose hydration. However, dried SPs exhibited no significant loss of mechanical integrity after repeated wet–dry cycles or exposure to aqueous media at pH 4 or 9, except under conditions causing cellulose depolymerization or silica dissolution. Short-term thermal aging at 100 °C for 1 week also had no measurable impact on their mechanical properties.

A broad range of particle types has been superstructured with nanofibers, and the resulting high cohesion is largely independent of particle identity. The ubiquitous presence of multiple interfibrillar interactions, even when the nanofiber fraction is rather low in the SPs (2–5 wt %), is far more relevant than interparticle interactions and drastically compensate for occasional poor particle–nanofiber interfaces (Figure 3a). Cellulose nanofibers have then been claimed as universal binders for particles. This universality may not be limited to cellulose nanofibers, and other nanofibers capable of nanonetworking may also display similar capabilities. As seen in Figure 3, CNFs have been used to bind particles of various surface chemistries (Figure 3b), sizes (Figure 3d), shapes (Figure 3e), dispersities (Figure 3f), complexities, including tubes, platelets 3D hedgehog-like, and living (Figure 3g), and natures, including metallic, ceramic, and polymeric (Figure 3h). Our pioneer, fundamental work on nanofiber-bound superstructures set a strong foundation for several advances in the area of particle–nanofiber materials, including cotton–pollen-based bioplastic,^29^ the use of nanofibers in sandy soil for strength and nutrient retention,^30^ superstructured particle fibers,^31^ as well as a whole new research field around amyloid-reinforced supraparticles for biomedical and pharmacy applications.^11^

## 4 Assembly Methods

Particle–nanofiber superstructures exhibit significantly greater robustness, attributed to the strong cohesion facilitated by the nanofiber networks.^4,6^ The macroscopic shapes of formed superstructures vary widely based on the assembly method used, including supraparticles, films, fibers, foams, and complex 3D-printed objects, which are discussed next (Figure 4a,b).

**Figure 4:**
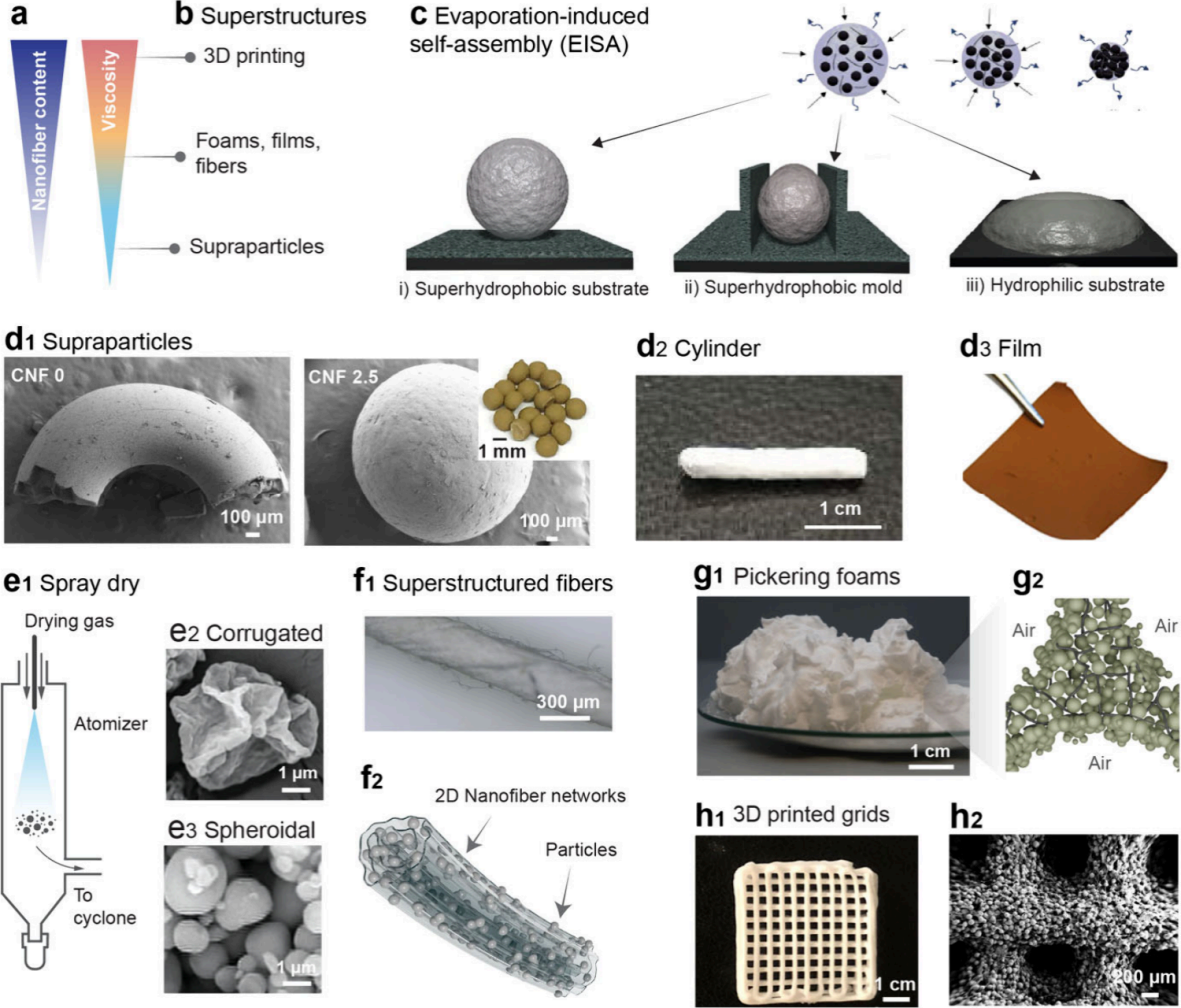
Superstructured constructs are fabricated through the assembly of nano/microparticles facilitated by nanofiber networks. (a) The incorporation of fibrillar biocolloids enables nanoparticles with tuned rheology and viscosity. (b) The various formulations enable a wide variety of superstructures. Reproduced with permission from ref 34. Copyright 2023 The Authors. (c, d) Evaporation-induced self-assembly (EISA) of aqueous suspensions results in highly cohesive particle–nanofiber superstructures. Reproduced with permission from ref 6. Copyright 2018 Wiley-VCH Verlag GmbH & Co. Additionally, panels (c, d) show assembly methods and photographs of superstructured spheres, films, and cylinders. Reproduced with permission from refs 6, 32, and 35. Copyright 2018 Wiley-VCH Verlag GmbH & Co, 2021 The Authors, and 2019 American Chemical Society. (e_1_) A schematic representation of the spray drying process. Scanning electron microscopy (SEM) images depict (e_2_) crumpled supraparticles and (e_3_) spheroidal supraparticles. Reproduced with permission from ref 11. Copyright 2024 Wiley-VCH Verlag GmbH & Co. (f_1_) Photograph of superstructured fibers and (f_2_) a schematic illustration showing two-dimensional cellulose nanosheets forming multilayer, roll-up structures, with nanoparticles encapsulated within the fibers. Reproduced with permission from ref 31. Copyright 2024 Springer Nature. (g_1_) Photograph of wet Pickering foams and (g_2_) a schematic illustration of the particle–nanofiber superstructures at the air–water interface. Reproduced with permission from ref 36. Copyright 2021 The Authors. (h_1_) Photograph of 3D-printed cubic grids and (h_2_) an SEM image illustrate the superstructured struts within these printed grids. Reproduced with permission from ref 37. Copyright 2019 Wiley-VCH Verlag GmbH & Co.

### 4.1 Supraparticles

The evaporation of aqueous suspensions at approximately 60 °C, when cast on superhydrophobic surfaces, typically resulted in isotropic consolidation (Figure 4c-i).^4,6,32^ The formation of a physically entangled cellulose nanofiber (CNF) network, even at low solid concentrations, was crucial for stabilizing the particle suspension. CNFs facilitate multiple physical interactions during drying-induced self-assembly, leading to a robust interlocked 3D network that physically reinforces the assembled supraparticles (SPs). During the drying process, the reduction of free water allowed the system to self-assemble into highly porous superstructures within 30 min. With CNF loadings exceeding 2.5 wt %, regular spherical SPs composed of closely packed nanoparticles were formed (Figure 4d_1_). In the absence of CNFs, the evaporation of particle suspensions resulted in torus-like superstructures that were prone to collapse during the drying process. Superstructured films were successfully obtained by casting the particle–CNF dispersions on hydrophilic substrates (Figure 4c-iii,d_3_). For more complex architectures, superhydrophobic molds were utilized, as demonstrated by assembly into cylindrical shapes (Figure 4c-ii,d_2_).

Spray drying particle dispersions containing amyloid nanofibers (ANFs) produces corrugated supraparticles (SPs; Figure 4e_1_,e_2_).^11^ ANFs promote the onset of buckling instabilities and, together with rapid consolidation during spray drying, induce structural arrest, creating corrugated SP morphologies. In contrast, spray drying pure particle suspensions yields noncorrugated SPs with toroidal, spheroidal, or spherical morphologies (Figure 4e_3_).^11,33^ The transition from noncorrugated to corrugated SPs occurs only when the fiber content increases by ∼1 vol % for every 10 nm increase in particle diameter. If the nanofiber fraction is below this linear threshold, noncorrugated SPs are formed, corresponding to low nanofiber contents.

### 4.2 Superstructured Films and Fibers

Superstructured films composed of lignin particles (LPs) are formed through a process of self-assembly, with CNFs serving as agents for network formation and cohesion (Figure 4d_3_).^35^ The addition of as little as 8 wt % CNFs significantly enhances the toughness and flexibility of these films. Although the superstructured film readily disintegrates in water, its water resistance can be improved by incorporating a wet-strength agent (WSA). Recently, this approach was extended to fabricate superstructured bioplastics from pollen particles and cotton microfibers.^29^ Under ambient air-drying, a cast slurry comprising defatted pollen particles and cotton microfibers self-assembled into a dense fiber-laminate bioplastic network. Composites with 30% fiber content exhibited the highest tensile strength (52.22 MPa) and Young’s modulus (2.24 GPa).

Superstructured nano/microfibers were produced by wrapping diverse powdery materials in two-dimensional (2D) cellulose, providing spatial confinement without compromising their native specialties and functionalities (Figure 4f_1_).^31^ When loaded with well-dispersed guest particles, the 2D cellulose nanosheets shrank and rolled at the solid–gas interface under contraction forces during lyophilization, ultimately forming multilayer roll-up structures that encapsulate the particles within the fiber (Figure 4f_2_). Customized fibers with a tunable diameter and particle loading can then be assembled into high-performance macroscopic materials with diverse geometries.

### 4.3 Superstructured Foams and Complex 3D Objects

Cellulose nanofibers (CNFs) can network around even PTFE particles. On that note, they are ideal for strengthening particle-stabilized foams by adding fibrillar networks around hydrophobic particles adsorbed at the air–liquid interface. Wet Pickering foams that are stabilized by hydrophobic silica particles networked with CNFs have exhibited long-term superstability, persisting for more than one year (Figure 4g_1_).^36^ In this case, the hydrophobic silica particles provided foaming by adsorbing at the air interfaces for the creation of pores, while CNFs reinforced and stabilized the air–water interface by particle–nanofiber networks (Figure 4g_2_). With 2–8 wt % CNFs, foamability increased by up to 350%. Upon drying, tight particle–nanofibril networks yielded cohesive superstructures that retained the shape of CNF-containing foam, whereas CNF-free foams collapsed within 5 min and lost their structure.

Superstructured inks have been formulated from suspensions of particles combined with cellulose nanofibers (CNFs), which function as binders and rheology modifiers, thereby inducing shear thinning properties. CaCO_3_ particles and nanofibers (5 wt %) have been used to engineer superstructured pastes that can be readily printed onto paper substrates to form fluidic channels.^38^ The fibrillar nanonetwork greatly improves the printability of CaCO_3_ particles while maintaining the excellent wicking properties. The flexible fluidic systems, with adjustable channels, hold promise for applications in flexible diagnostics and electronics. Black bioinks have been produced from aqueous colloidal suspensions of light-absorbing carbon microspheres and CNFs.^34^ Upon consolidation, particle–fiber nanonetworks can develop nanoscale structural features and reduce the packing density of solid constructs, thereby inducing light entrapment. Additionally, particle–nanofiber constructs facilitate the adhesion of bioinks to various surfaces, such as paper, wood, and textiles. Superstructured inks could be used to create objects showing high levels of geometrical complexity at the millimeter scale (Figure 4h_1_,h_2_).^4,34,37^ The printed struts exhibit clear boundaries, remaining separate even after drying, as the particles are integrated within the fiber networks. 3D printing creates particle-based objects featuring customized microstructures, thereby expanding the potential for applications that require complex constructs.

## 5 Exploiting Supraparticle Designs in Practical Applications

Coassembly of different building blocks preserves their functionalities, such as bioactivity, the biological machinery of cells, and magnetism.^39–41^ SPs confine a large number of particles in the fibrillar network, which are considerably larger than their solution-phase, emulsion-templated counterparts,^39^ enabling emergent functionalities.

### 5.1 Cargo Loading, Protection, and Controlled Release

Cargo loading is a crucial method for modulating the functions of supraparticles (SPs), as a carrier.^6^ For sintered SPs, temperature-sensitive cargo can be loaded only via postloading. In the case of CNF-bound SPs, temperature-sensitive cargo can be introduced using postloading, preloading, or *in situ* loading strategies (Figure 5a). The incorporation of the model cargo, whether through preloading or postloading, only slightly reduces the mechanical properties of the biogenic silica SPs. The release profiles of SPs loaded with biocide were comparable to those of biogenic silica nanoparticles, highlighting the preserved surface area of the nanoparticles and the easy access of solvents to their surface. However, embedding biocide-loaded silica nanoparticles in CNF-bound nanocomposites substantially reduces their release rate compared to silica nanoparticles.^42^

**Figure 5:**
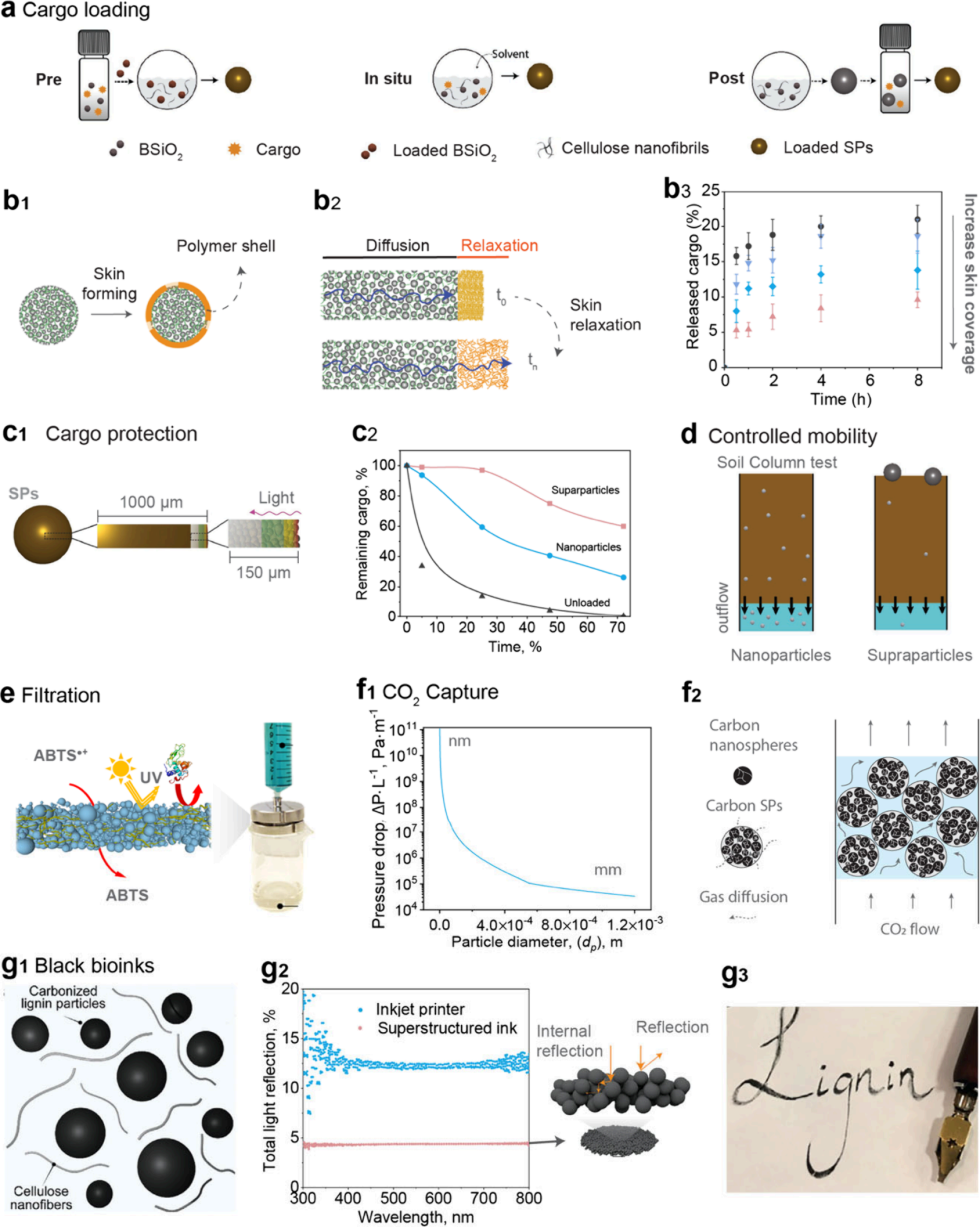
(a) Schematic illustration of three distinct cargo loading methodologies used to assemble the biobased biocidal superstructures. Reproduced with permission from ref 6. Copyright 2018 Wiley-VCH Verlag GmbH & Co. Schematic (not to scale) illustrating (b_1_) the skin formed on the surface of printed struts and (b_2_) the drug-release pathway. (b_3_) Release profiles of sodium salicylate loaded into CNF-bound silica particles. Reproduced with permission from ref 37. Copyright 2019 Wiley-VCH Verlag GmbH & Co. (c_1_) Schematic illustration of zones within a supraparticle subject to photodegradation. (c_2_) Photodegradation kinetics of unloaded thymol and thymol encapsulated in SPs under UV exposure (λ_max_ = 356 nm). (d) Schematic illustrating the mobility of nanoparticles and SPs in simulated soils. Reproduced with permission from ref 6. Copyright 2018 Wiley-VCH Verlag GmbH & Co. (e) Lignin-based membranes have been evaluated for active filtration applications. Reproduced with permission from ref 35. Copyright 2019 American Chemical Society. (f_1_) Theoretical pressure drop per unit length (Δ*P*·*L* ^–1^) in a packed column as a function of particle diameter (*d*_*p*_). (f_2_) Schematic illustrating that carbon SPs can enhance gas dynamics within a packed column. Reproduced with permission from ref 32. Copyright 2021 The Authors. (g_1_) Black bioinks are prepared using carbonized lignin particles (LPs) and CNFs. (g_2_) Total light reflection of the inkjet-printed black color and the dried black bioinks. (g_3_) Demonstration as inks for use in dipping pens. Reproduced with permission from ref 34. Copyright 2023 The Authors.

3D printing of superstructured ink enables the creation of grid-like structures with skin-bearing architectures at the micrometer scale, facilitating precise control over the release of loaded cargo (Figure 5b_1_,b_2_).^37^ Although the release profiles of printed grids with varying levels of skin coverage exhibited similar shapes, remarkable control was achieved: increased skin coverage gradually slowed the release rates (Figure 5b_3_). This approach allows for a high degree of topological complexity and precise control over drug release by combining pore diffusion with skin relaxation. The nanofiber superstructures formed under ambient or mildly elevated temperatures and are compatible with retaining metabolic activity in living systems.^4^ For example, superstructured living yeast particles preserved their metabolic functions and facilitated the rapid exchange of nutrients and metabolic products in and out of the SPs. During fermentation, yeast particles that are immobilized by the entangled network produce CO_2_ and ethanol, which are released from the SPs at rates depending on the availability of sugars such as sucrose and glucose.

Supraparticles (SPs) enhance the protection of encapsulated cargo as light penetrates only the outermost 150 μm of the entire sphere (Figure 5c_1_).^6^ SPs with a diameter of ∼2 mm provided ∼85% protection of the cargo from visible light exposure and 65% protection from UV irradiation, representing at least a 2-fold increase in retention compared to nanoparticles (Figure 5c_2_). Thus, SPs could effectively preserve the long-term bioactivity or bioavailability of given cargos. SPs also play a crucial role in mitigating the negative environmental impacts associated with the high mobility of nanoparticles (Figure 5d).^6^ Nanoparticles exhibit very high permeability in soil, leading to either partial accumulation within the soil matrix or rapid diffusion to the outflow. In addition to their potential for bioaccumulation, nanoparticles can act as carriers for pesticides, posing a risk of groundwater contamination. In contrast, SPs display markedly limited mobility in model soils, with penetration restricted to just a few millimeters beneath the surface. The findings also indicate that less than 5% of SPs are diffused to the outflow compared to more than 95% for nanoparticles.

### 5.2 Superstructured Adsorbents and Light Superabsorbers

Superstructured films, containing up to 92% lignin particles (LPs) and CNFs as a binder, reveal a homogeneous pore structure with a narrow size distribution (Figure 5e).^35^ At pressures over 80 kPa, these LP-based films demonstrate higher water permeability than Whatman filter paper, suitable for micro- and nanofiltration membranes in crossflow configurations. These superstructured membranes also retain intrinsic lignin features such as antioxidation, UV blocking, reduced surface energy, and antifouling capabilities.

Lignin SPs were converted into robust carbon SPs through oxidative stabilization followed by carbonization.^32,43^ Their high cohesion is mainly attributed to the partially melted LPs creating an interparticle network during carbonization. The robust carbon SPs with a “particle-of-particles” structure afforded easy handling as adsorbents for CO_2_ capture. For instance, given their high strength, a close-packed bed of such carbon SPs could theoretically reach a maximum height of 1218 m. The normalized pressure loss (Δ*P*·*L* ^–1^) of a flue gas flowing through a column filled with carbon SPs was significantly lower than that observed with nanoparticles (Figure 5f_1_). The carbon SPs can adsorb 78 mg CO_2_·g^–1^, demonstrating a promising method for carbon sequestration (Figure 5f_2_). Still on carbon-based superstructures, it has been demonstrated that coatings made only of submicron particles are brittle, and they tend to self-organize into ordered packing upon consolidation from aqueous colloidal suspensions.^34^ However, superstructuring particles with CNFs disrupts the ordered packing and creates an irregular and porous particle network, inducing multiple internal light reflections (Figure 5g_1_,g_2_). Consolidation of the superstructured black inks results in particle–nanofiber networks, achieving a low light reflectance of ∼4%, which have been applied as inks in dipping pens for writing (Figure 5g_3_).

## 6 Outlook and Future Directions

A broad range of nanofiber parameters should be elucidated to push forward nanofiber–particle assemblies. The intrinsic mechanical properties and surface features of nanofibers should play a major role in how they conform around particles, as well as how they transfer cohesion from the single building block level to macroscopic superstructured constructs. This is highly relevant when broadening this concept to other nanofibers, such as chitinous,^12^ ceramic,^44^ and aramid nanofibers,^45^ something that cellulose nanofibers likely do not excel in. Next, the main challenges and open questions in the field are detailed:

(1) Limited understanding of particle–nanofiber behavior.
  Computational modeling and mathematical models can help to explain the assembly behavior and dynamics of nanofiber–particle systems. For particle-only supraparticles, simulations of particle behavior during consolidation have provided an insightful understanding about superstructuring and mechanical properties of supraparticles.^7,46^ On the other hand, there are several analytical models to explain fiber and nanofiber networks, for example, the crowding factor.^47^ New crowding factors have been derived that account for the volume occupied by particles around nanofibers, providing improved insight into nanofiber populations in the presence of particles and enabling the estimation of particle–nanofiber contacts.^4^ Graph theory could be used to better understand these complex networks, as it has been elegantly used to study nanofiber networks.^48^
(2) Production rate and scaling up.
  For large-scale manufacturing, supraparticle manufacturing could be improved by implementing automated and robotic casting lines. Supraparticle fabrication methods are yet an area for development, and speeding up the consolidation as well as increasing its scale are central to define commercial inroads for such materials. Other techniques such as emulsion templating, spray drying, and microfluidics could help to speed up the fabrication of tunable particle–nanofiber materials. Upcoming studies on larger-scale supraparticle fabrication could benefit from the recent advances in parallel fields, such as spray-assisted fabrication of structured pigments^49^ and 4D printing.^50^
(3) Broadening supraparticle applications.
  Research on particle superstructuring has become relevant for several emerging areas, such as colloidal robotics^51^ and optofluidics.^52^ Nanoparticles are superstructured to build constructs at nanoliter volumes for sensing, computation, communication, and locomotion.^51^ Supraparticles can be designed to behave collectively, as colloidal robots, by following optofluidic fabrication methods.^52^ It is expected that the localized assembly of particles within the nanofibrillar network can confine their functions and thereby contribute to the emergent behaviors in colloidal robotics.
